# Changing Clinical Spectrum of Invasive Meningococcal Disease in France (2014–2025): Impact of Age and Meningococcal Lineage on Atypical Presentations

**DOI:** 10.3390/microorganisms14020356

**Published:** 2026-02-03

**Authors:** Samy Taha, Ala-Eddine Deghmane, Muhamed-Kheir Taha

**Affiliations:** Invasive Bacterial Infections Unit, National Reference Centre for Meningococci and Haemophilus Influenzae, Institut Pasteur, Université Paris Cité, 28 rue du Dr Roux, CEDEX 15, 75724 Paris, France

**Keywords:** invasive meningococcal disease, atypical clinical presentations, bacteraemic pneumonia, abdominal manifestations, serogroups W and Y, clonal complexes, early mortality, antimicrobial susceptibility

## Abstract

Invasive meningococcal disease (IMD) is classically associated with meningitis and septic shock, but an increasing proportion of cases present with atypical, extra-meningeal manifestations. Following the COVID-19 pandemic, major epidemiological shifts have occurred in France, including a rebound in IMD incidence and changes in circulating serogroups and clonal complexes. We conducted a nationwide retrospective study including all laboratory-confirmed IMD cases analysed by the French National Reference Centre between July 2014 and June 2025. Clinical presentations were coded as non-exclusive entities. Associations with age, serogroup, clonal complex, antimicrobial susceptibility and early mortality (≤72 h) were assessed using descriptive analyses and multivariable logistic regression models. Among 4328 IMD cases, sepsis/shock (61.1%) and meningeal involvement (54.9%) predominated, while atypical forms were frequent, including bacteraemic pneumonia (7.7%), abdominal presentations (8.0%) and arthritis (6.0%). Bacteraemic pneumonia was strongly associated with older age and serogroups W and Y, whereas abdominal forms predominated in adolescents and young adults and were independently associated with serogroups W and Y and clonal complex (cc) cc11. Abdominal presentations were independently associated with early mortality (adjusted odds ratio [aOR] 2.40) but not meningococcal pneumonia. Abdominal presentations were associated with serogroup W (aOR 2.27; 95% CI 1.35–3.83) and serogroup Y (aOR 2.92; 95% CI 1.79–4.75) and with cc11 (aOR 1.77; 95% CI 1.07–2.94). In contrast, cc23 was associated with lower odds of abdominal involvement (aOR 0.42; 95% CI 0.25–0.70). Overall, atypical presentations now represent a substantial proportion of IMD in France and are strongly shaped by age and meningococcal lineage. These findings highlight diagnostic challenges, prognostic heterogeneity and the need for continued integrated clinical, microbiological and genomic surveillance in the context of evolving vaccination strategies.

## 1. Introduction

Invasive meningococcal disease (IMD) is a rapidly progressive and potentially fatal infection caused by *Neisseria meningitidis*. It is classically perceived as fulminant meningitis or septic shock with purpura fulminans, reflecting its most dramatic and well-recognised manifestations [1,2].

IMD is commonly described using two complementary frameworks. A first classification is microbiological and relies on capsular serogroup, which underpins surveillance, vaccination strategies and population dynamics. A second, clinically oriented classification is based on disease presentation. Our study aims to link both. The international literature indicates that IMD displays a broader clinical spectrum than traditionally recognised. Several reviews acknowledge the existence of extra-meningeal manifestations, including bacteraemic pneumonia, arthritis, pericarditis and abdominal syndromes [3,4]. However, these extra-meningeal manifestations are rarely quantified in population-based datasets, limiting our understanding of their frequency and lineage-specific determinants [4,5,6].

Beyond serogroup, meningococcal population structure is further characterised by clonal complexes (CCs), defined by multilocus sequence typing. A limited number of hyperinvasive CCs account for most IMD cases worldwide and are known to differ in age distribution, epidemic potential and clinical expression [7].

In France, two pre-COVID investigations provided the first structured descriptions of atypical IMD forms. Vienne et al. reported 33 bacteraemic pneumonias and 26 arthritis cases between 1999 and 2002, mostly associated with serogroup W and affecting older adults [8]. Later, Guiddir et al. identified 105 abdominal presentations between 1991 and 2016 (approximately 1% of IMD) but showed an increasing trend since 2014, predominantly in younger patients and with markedly higher case-fatality rates, largely linked to the serogroup W of South American–UK lineage [9]. Although these studies confirmed that atypical presentations were historically rare, they preceded the profound epidemiological and microbiological shifts observed during and after the COVID-19 pandemic [10,11].

More recent international evidence suggests that atypical forms may be more common than previously recognised, particularly among infections caused by serogroups W and Y [12,13,14,15,16,17]. In England, pneumonic presentations accounted for 12–26% of MenW and MenY infections, while gastrointestinal symptoms occurred in up to 30% of MenW cases, especially among older adults [18,19,20].

To update the national situation, we recently analysed IMD cases from 2015 to 2022 and documented a rise in atypical presentations with age- and serogroup-specific patterns [21]. However, this period ended just as IMD incidence sharply rebounded in late 2022, driven by expanding clonal complexes such as cc9316, and before the further increase reported in 2024–2025 [22]. In addition, antimicrobial susceptibility data were not assessed, and multivariable modelling was not performed. Increasing reports of *N. meningitidis* strains with reduced susceptibility to beta-lactams further underscore the need to integrate phenotypic resistance into the clinical epidemiology [23]. Integrating minimal inhibitory concentration (MIC) data is increasingly relevant for stewardship and clinical guidelines, yet the clinical-phenotypic correlates of MIC patterns remain largely unexplored.

Despite these contributions, important epidemiological gaps therefore remain. Previous French and international surveillance studies have generally described IMD trends by age and serogroup but have rarely quantified the full burden of extra-meningeal presentations at a national scale while simultaneously integrating meningococcal lineage (clonal complexes), antimicrobial susceptibility and early mortality in a multivariable framework. Moreover, most available analyses predate the post-2022 rebound and the rapid expansion of emerging clonal complexes, limiting their ability to capture recent shifts in clinical phenotypes. As a result, the independent determinants of atypical IMD presentations and their prognostic implications in the current epidemiological context remain insufficiently characterised.

This study aimed to describe the national clinical spectrum of IMD in France (2014–2025) and identify independent associations with age, serogroup, clonal complex and antimicrobial susceptibility, including determinants of early mortality.

## 2. Materials and Methods

### 2.1. Study Design and Data Sources

This retrospective study included all laboratory-confirmed invasive meningococcal disease (IMD) cases analysed by the French National Reference Centre for Meningococci and *Haemophilus influenzae* (NRCMHi) between 1 July 2014 and 30 June 2025 as part of its mission in national epidemiological surveillance. IMD is subject to mandatory notification in France according to a standardised case definition [10]. Hospitals routinely send clinical samples from suspected cases (blood, cerebrospinal fluid (CSF), or other normally sterile sites) to the NRCMHi for confirmation and characterisation.

For each suspected IMD case, clinicians complete a standardised notification form documenting demographic information, initial clinical presentation and short-term outcome. These data are reviewed and updated during telephone feedback with treating clinicians when laboratory results are returned, ensuring that clinical information is consistently validated and harmonised for surveillance.

The analytic dataset included age, sex, epidemiological year (July–June), clinical presentation(s), early outcome, serogroup, clonal complex and antimicrobial susceptibility results.

### 2.2. Case Definition

Biologically confirmed IMD was defined as the detection of *N. meningitidis* in a normally sterile site by culture and/or PCR. Early death was defined as death occurring 72 h or less after initial clinical presentation or hospital admission.

### 2.3. Clinical Presentation Definitions

Because IMD can involve multiple organ systems, each case could fulfil several non-exclusive clinical presentations. Definitions were adapted from standard NRCMHi criteria and previous literature [4].

Sepsis or septic shock was defined as meningococcal bacteraemia associated with systemic inflammatory features, including purpura fulminans or other purpuric lesions, without another primary focus.

Meningeal form was defined as isolation of *N. meningitidis* from CSF and/or blood together with CSF leukocytosis greater than 10 cells per microlitre and compatible symptoms, including headache, vomiting, neck stiffness, photophobia or altered mental status.

Bacteraemic pneumonia was defined as meningococcal bacteraemia with fever, respiratory symptoms such as cough, dyspnoea or pleuritic pain and radiological evidence of pneumonia.

Abdominal form was defined as meningococcal bacteraemia with acute abdominal pain, diarrhoea or gastroenteritis-like illness in the absence of another enteric pathogen; meningococcal peritonitis was included.

Arthritis was defined as isolation of *N. meningitidis* from joint fluid or blood in acute mono- or oligo-arthritis confirmed clinically or by imaging.

Epiglottitis was defined as fever with odynophagia, dysphagia, dysphonia or inspiratory stridor, supported when available by laryngoscopic or imaging evidence of epiglottic oedema, and meningococcal isolation from blood or upper-airway specimens.

Cardiac forms included pericarditis, endocarditis or myocarditis with detection of *N. meningitidis* in blood, pericardial fluid or cardiac tissue, supported by echocardiographic or electrocardiographic abnormalities.

Rare presentations such as endophthalmitis, necrotising fasciitis and chronic meningococcaemia were recorded but excluded from regression analyses due to very small numbers.

### 2.4. Data Quality, Clinical Ascertainment and Potential Misclassification

Clinical information was collected and coded using the standardised national notification form of the French National Reference Centre, completed at admission by the reporting clinical team and reviewed at microbiological confirmation. This procedure, combined with routine feedback to reporting centres, contributes to the harmonisation of clinical data collection over time and across sites.

Clinical presentations were coded using predefined operational definitions and were treated as non-exclusive entities to reflect the multisystem nature of invasive meningococcal disease. To minimise misclassification with more prevalent non-meningococcal conditions, atypical presentations were defined conservatively as systemic illness with microbiologically confirmed *N. meningitidis* infection from a sterile site, combined with compatible organ-specific features (e.g., radiological evidence of pneumonia for bacteraemic pneumonia; acute abdominal pain and/or diarrhoea or gastroenteritis-like symptoms for abdominal presentations), in the absence of an alternative documented pathogen when available.

Although symptom ascertainment for subjective manifestations such as abdominal pain may vary between clinicians and centres, all cases included in the analysis required laboratory confirmation of invasive meningococcal infection, which substantially limits diagnostic misclassification. Potential residual heterogeneity is addressed in the Discussion.

### 2.5. Microbiological Procedures

Isolates were identified and characterised using standard procedures routinely applied at the French NRCMHi [10]. Minimal inhibitory concentrations (MICs) for penicillin G, amoxicillin, cefotaxime, rifampicin, ciprofloxacin and azithromycin were determined using the Etest gradient diffusion method (bioMérieux, Marcy-l’Étoile, France). Interpretations followed the NRCMHi routine interpretation framework, which aligned with the recommendations of the European Meningococcal and *Haemophilus influenzae* Society for penicillin G and amoxicillin and the European Committee on Antimicrobial Susceptibility Testing for the other tested antibiotics [24].

For penicillin G and amoxicillin, isolates with MICs greater than or equal to the breakpoints of 0.125 mg per litre and 0.250 mg per litre, respectively, were categorised as non-susceptible, including intermediate and resistant categories [23]. Beta-lactamase activity was assessed using a chromogenic cephalosporin test (Cefinase™, Becton Dickinson, Sparks, MD, USA).

Penicillin G non-susceptibility was historically first used and therefore selected as the primary resistance outcome because it was the most frequently observed and consistently documented phenotype over the study period, reflecting well-characterised alterations of the *penA* gene rather than high-level resistance [25].

### 2.6. Variable Recoding

Serogroups were grouped as B, C, W, Y, X or Other. Clonal complexes were recoded into nine categories: cc11, cc9316, cc32, cc23, cc269, cc162, cc213, cc461 and Other or non-assigned. Age was categorised into nine groups: less than 1 year, 1 to 4, 5 to 9, 10 to 14, 15 to 19, 20 to 24, 25 to 44, 45 to 64 and 65 years or older. Clinical forms were treated as non-exclusive binary variables.

For descriptive analyses, all cases were classified as early death or non-death, defined as alive more than 72 h or unknown. For multivariable modelling of early death, only cases with documented outcomes were retained and cases with unknown outcomes were excluded. Early outcome was documented for 870 cases.

### 2.7. Statistical Analyses

Distributions of demographic, microbiological and clinical variables were described using counts and percentages. Associations between clinical forms and categorical variables, including serogroup, age group, epidemiological year, clonal complex and early death, were assessed using Pearson’s chi-squared test; Fisher’s exact test was used for tables with expected counts less than five. Non-susceptibility proportions were compared between cases with and without each clinical form using the same approach. Given the large sample size and the number of comparisons, *p*-values were interpreted alongside effect sizes and consistency across analyses.

Separate multivariable logistic regression models were fitted for sepsis or shock, meningeal form, bacteraemic pneumonia, abdominal forms and arthritis. Covariates were age group, sex, serogroup and clonal complex, with the following reference categories: less than 1 year, female sex, serogroup B and clonal complex Other or non-assigned. All covariates were included a priori based on biological plausibility and established epidemiological evidence, and no automated selection procedure was used.

Separate multivariable logistic regression models were fitted for each major clinical presentation because clinical forms were not mutually exclusive and frequently co-occurred. Multinomial modelling frameworks are not appropriate under these conditions, as they assume mutually exclusive outcome categories. Modelling each presentation as a binary outcome allows estimation of clinically interpretable associations (odds of presenting with a given syndrome) while adjusting for shared determinants.

Age, serogroup and clonal complex were included simultaneously in multivariable models to account for their inter-relationships. Although serogroup and clonal complex are biologically related, they are not redundant, as multiple clonal complexes circulate within the same serogroup and several clonal complexes have been observed across different serogroups over time. Both variables were therefore retained a priori to disentangle capsule-associated from lineage-associated effects. Model stability was assessed through coefficient estimates and confidence intervals, and no evidence of instability or convergence issues was observed in the main analyses.

Given the potential biological correlation between meningococcal serogroups and clonal complexes, collinearity between these variables was formally assessed. A strong association was observed (Cramer’s V = 0.56), but variance inflation factors remained below critical thresholds (maximum VIF < 4), and model condition numbers indicated acceptable multicollinearity. Both variables were therefore retained in adjusted models to allow separation of capsule-related and lineage-specific effects.

Early death was analysed using a logistic regression model restricted to cases with known outcome, adjusting for age group, sex, serogroup, clonal complex and major clinical forms. Determinants of penicillin G non-susceptibility, defined as MIC greater than or equal to 0.125 mg per litre, were evaluated using separate models with the same reference categories, with and without adjustment for clinical forms.

Analyses were performed using Biopython (v3.13.9; Python Software Foundation, Wilmington, DE, USA) [26], including the pandas (v2.3.3; pandas developers, online) and statsmodels (v0.14.5; statsmodels developers, online) libraries. Analyses were run in Jupyter Notebook (v7.4.5; Project Jupyter, online) within the Anaconda distribution (Anaconda, Inc., Austin, TX, USA) on macOS (Apple Inc., Cupertino, CA, USA). A two-sided *p*-value less than 0.05 was considered statistically significant.

### 2.8. Ethics and Funding

These data were included anonymously in the database after excluding personal data as part of the mission of the National Reference Centre for meningococci and *Haemophilus influenzae* for routine surveillance of IMD and isolate identification and typing. The procedure for collecting samples and information was submitted and approved by the Commission Nationale de l’Informatique et des Libertés under approval number 1475242 of 2011, and the requirement for consent was waived. The study was funded by the Institut Pasteur and Santé Publique France. Both funders had no role in designing, conducting, analysing or writing the study. Study design, data collection, data analysis, data interpretation and writing of this report were performed by the authors.

## 3. Results

### 3.1. Overall Characteristics of the Study Population

From July 2014 to June 2025, 4328 confirmed IMD cases were included.

Serogroup B was the most frequent (n = 2067; 47.8%), followed by W (n = 846; 19.5%), Y (n = 780; 18.0%), C (n = 565; 13.1%), X (n = 22; 0.5%) and other/non-groupable (48; 1.1%).

The age distribution showed the three peaks pattern: infants < 1 year accounted for 12.9% (n = 558) of cases and adults ≥ 65 years for 21.6% (n = 933), with intermediate peaks in adolescents and young adults (15–24 years: n = 857; 19.8%). One case had a missing age. Baseline demographic and microbiological characteristics are summarised in Table 1.

### 3.2. Frequency of Clinical Presentations

Clinical forms were non-exclusive. Overall, sepsis or septic shock was the most frequent presentation (n = 2645; 61.1%), followed by meningeal involvement (n = 2375; 54.9%).

**Figure 1:**
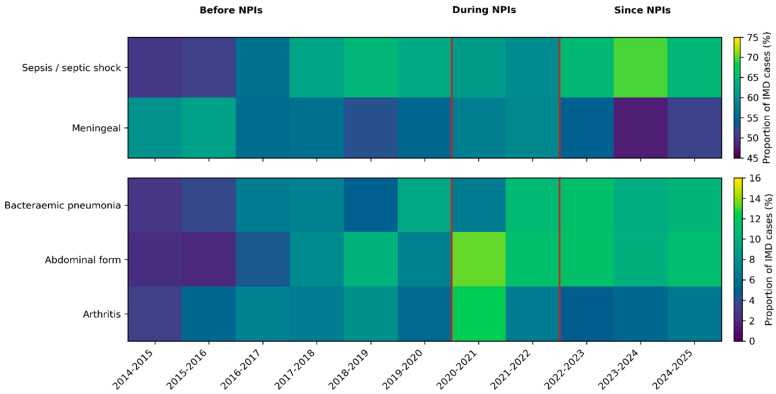
Temporal evolution of major clinical presentations of invasive meningococcal disease in France, 2014–2025. Proportions of invasive meningococcal disease (IMD) cases presenting with sepsis/septic shock, meningeal involvement, bacteraemic pneumonia, abdominal forms and arthritis across epidemiological years (July–June). Heatmaps display the percentage of IMD cases with each non-exclusive clinical presentation. Vertical red lines delineate periods before, during and after the implementation of non-pharmaceutical interventions (NPIs) related to the COVID-19 pandemic.

Among atypical forms, bacteraemic pneumonia occurred in 334 cases (7.7%), abdominal presentations in 345 (8.0%) and arthritis in 258 (6.0%). Epiglottitis and cardiac forms were rare, with 20 (0.5%) and 38 (0.9%) cases, respectively. Temporal changes in the distribution of major clinical presentations across epidemiological years, including the periods before, during and after non-pharmaceutical interventions related to the COVID-19 pandemic, suggest an increasing trend of atypical presentations, including meningococcal pneumonia and abdominal forms, after non-pharmaceutical interventions (Figure 1).

### 3.3. Associations Between Clinical Presentations and Serogroup

All clinical forms were significantly associated with serogroup (global chi-squared tests, all *p* ≤ 2 × 10^−5^), including the rare forms such as epiglottitis and cardiac involvement (*p* = 1.9 × 10^−5^ and 1.9 × 10^−7^, respectively).

Bacteraemic pneumonia was strikingly enriched in serogroups Y and W: it represented 18.8% of IMD due to Y and 15.1% of IMD due to W, compared with 1.6% among serogroup B cases. Abdominal forms were likewise more frequent in W (15.7%) and Y (8.6%) than in B (5.4%). Arthritis occurred proportionally more often in serogroups C (approximately 10%) and W (approximately 8%), while epiglottitis was predominantly associated with serogroup W (approximately 1.5%).

**Figure 2:**
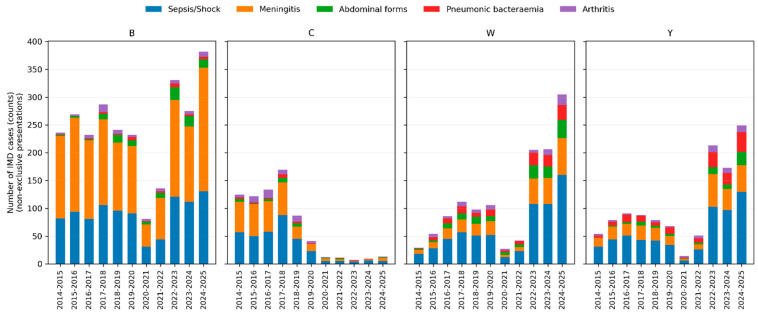
Clinical presentations of invasive meningococcal disease by serogroup and epidemiological year. Stacked bar charts showing the number of IMD cases with each non-exclusive clinical presentation according to epidemiological year, stratified by serogroup (B, C, W and Y). Bars represent absolute counts of cases; colours indicate sepsis/septic shock, meningeal presentation, abdominal form, bacteraemic pneumonia and arthritis.

Conversely, the meningeal form was most frequent in serogroup B (72.7%) and less common in W and Y (approximately 33–36%). The distribution of clinical presentations by serogroup across epidemiological years is illustrated in Figure 2.

### 3.4. Associations with Age Group

Age group was strongly associated with all clinical forms (chi-squared tests, all *p* ≤ 0.02; *p* = 0.013 for cardiac forms).

Bacteraemic pneumonia displayed a marked age gradient: it was rare in children (<1 year: 0.7% of IMD; 1–4 years: 0.4%) but increased steadily with age, reaching 6.7% in adults 45–64 years and 25.4% in those ≥65 years.

**Figure 3:**
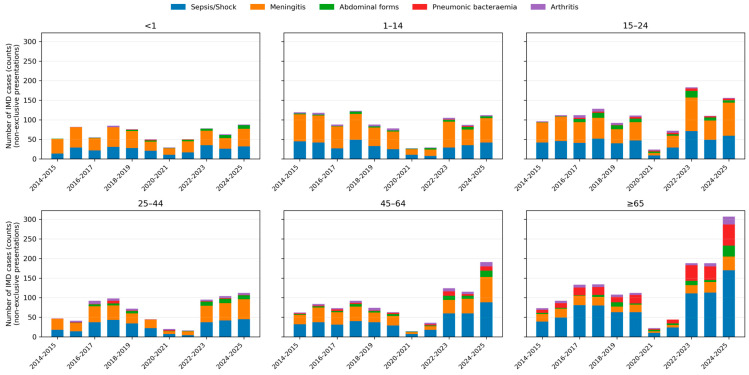
Age-specific distribution of clinical presentations of invasive meningococcal disease over time. Stacked bar charts depicting the number of IMD cases with each non-exclusive clinical presentation across epidemiological years, stratified by age group (<1, 1–14, 15–24, 25–44, 45–64 and ≥65 years). Bars represent absolute counts; colours indicate sepsis/septic shock, meningeal involvement, abdominal forms, bacteraemic pneumonia and arthritis.

Abdominal presentations peaked in adolescents and young adults, with proportions of approximately 9–10% in the 15–24-year age group, and remained around 8–9% in older adults. Meningeal presentations dominated in children and young adults, whereas sepsis or shock was particularly frequent among older adults. Age-specific patterns and temporal trends in clinical presentations are shown in Figure 3.

### 3.5. Temporal Trends

When stratified by epidemiological year, the distributions of sepsis or shock, meningeal disease, bacteraemic pneumonia and abdominal forms all showed significant heterogeneity over time (chi-squared *p* ≤ 10^−3^), suggesting temporal changes in the spectrum of disease presentations. Cardiac forms and arthritis did not show significant temporal variation (*p* = 0.73 and *p* = 0.06, respectively) (Figure 1).

### 3.6. Associations with Clonal Complex

Clonal complexes were significantly associated with all clinical forms (global chi-squared tests, all *p* < 0.01, and *p* < 10^−11^ for sepsis or shock, meningitis, pneumonia and abdominal forms). This observation is consistent with the circulation of hyperinvasive lineages [7].

Bacteraemic pneumonia was particularly frequent in cc23 (18.6%), cc9316 (10.9%) and cc11 (10.0%), compared with 2% or less in cc32, cc162 or cc269.

Abdominal presentations were enriched in cc11 (11.7%) and cc9316 (12.6%), while arthritis occurred more often in cc11 (8.8%), cc23 (7.6%) and cc9316 (6.3%).

**Figure 4:**
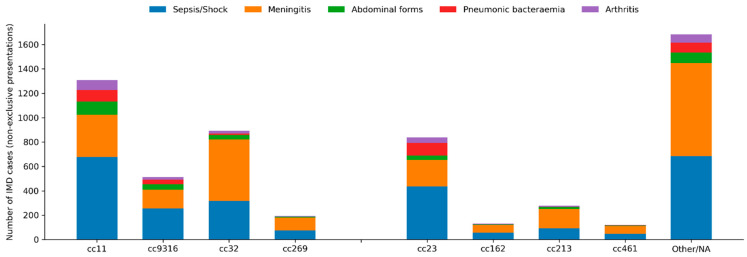
Distribution of clinical presentations of invasive meningococcal disease according to clonal complex. Stacked bar chart showing the cumulative number of IMD cases with each non-exclusive clinical presentation according to clonal complex over the entire study period (2014–2025). Bars represent absolute counts; clinical presentations include sepsis/septic shock, meningeal involvement, abdominal forms, bacteraemic pneumonia and arthritis. “Other/NA” includes rare or unassigned clonal complexes.

Epiglottitis was mainly observed in cc11 and cc23. The distribution of clinical presentations according to clonal complex is presented in Figure 4.

### 3.7. Early Mortality

Overall, 328 of 4328 IMD cases (7.6%) were classified as early deaths occurring within 72 h, while 4000 cases (92.4%) did not die within this timeframe, including cases with unknown short-term outcome.

In univariable analyses using all cases, early death was more frequent among patients with sepsis or shock than among those without (277 of 2645; 10.5% versus 51 of 1683; 3.0%; chi-squared *p* = 3.3 × 10^−19^). It was also more common in bacteraemic pneumonia (40 of 334; 12.0% versus 288 of 3994; 7.2%; *p* = 2.3 × 10^−3^) and particularly in abdominal forms (51 of 345; 14.8% versus 277 of 3983; 7.0%; *p* = 2.4 × 10^−7^).

Conversely, early death was less frequent among cases with meningeal presentation (115 of 2375; 4.8% versus 213 of 1953; 10.9%; *p* = 9.8 × 10^−14^) and arthritis (1 of 258; 0.4% versus 327 of 4070; 8.0%; *p* = 1.2 × 10^−5^). No significant association was observed for epiglottitis (*p* = 0.39) or cardiac forms (*p* = 1.00). Multivariable analysis suggested that early death remained independently associated with sepsis or shock (adjusted odds ratio 2.48; 95% confidence interval 1.64–3.73) and abdominal presentations (adjusted odds ratio 2.40; 95% confidence interval 1.41–4.10). In contrast, meningeal presentations (adjusted odds ratio 0.92; 95% confidence interval 0.63–1.33) and bacteraemic pneumonia (adjusted odds ratio 0.80; 95% confidence interval 0.46–1.39) were not significantly associated with early mortality after adjustment.

Among serogroups, serogroup W showed a strong independent association with early death (adjusted odds ratio 4.30; 95% confidence interval 2.03–9.09), whereas other serogroups did not. Age and sex were not significantly associated with early death in this adjusted model.

### 3.8. Multivariable Analysis of Determinants of Clinical Forms

**Figure 5:**
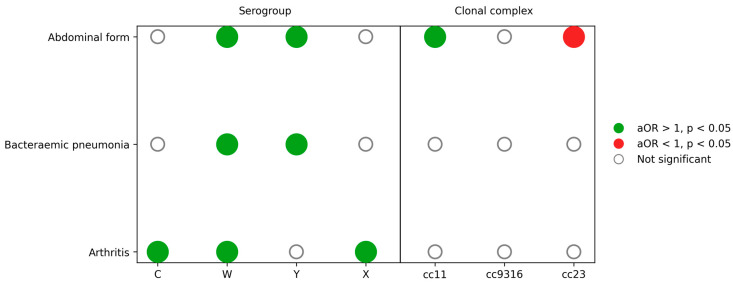
Independent associations between atypical clinical presentations and meningococcal serogroups and clonal complexes. Matrix diagram summarising adjusted associations from multivariable logistic regression models for abdominal forms, bacteraemic pneumonia and arthritis. Dots represent adjusted odds ratios (aORs) for each serogroup or clonal complex compared with the reference categories (serogroup B and Other/NA clonal complex). Green dots indicate aOR > 1 with *p* < 0.05, red dots indicate aOR < 1 with *p* < 0.05 and empty circles indicate non-significant associations. Models were adjusted for age group, sex, serogroup and clonal complex as appropriate.

Multivariable logistic regression models were fitted separately for abdominal forms, bacteraemic pneumonia, sepsis or shock, meningeal presentations and arthritis, adjusting for age group, sex, serogroup and clonal complex. In adjusted analyses, age and serogroup were the dominant determinants of clinical forms, with a striking age gradient for bacteraemic pneumonia (65 years or older: adjusted odds ratio 24.1) and markedly lower odds of meningitis for serogroups W and Y compared with B. Adjusted determinants of atypical clinical presentations (abdominal form, bacteraemic pneumonia and arthritis) are detailed in Table 2 and summarised in Figure 5.

Abdominal presentations remained independently associated with serogroup W (adjusted odds ratio 2.27; 95% confidence interval 1.35–3.83) and serogroup Y (adjusted odds ratio 2.92; 95% confidence interval 1.79–4.75) and with cc11 (adjusted odds ratio 1.77; 95% confidence interval 1.07–2.94) but not with cc23 (adjusted odds ratio 0.42; 95% confidence interval 0.25–0.70).

Bacteraemic pneumonia was strongly and independently associated with serogroup W (adjusted odds ratio 5.14; 95% confidence interval 2.68–9.86) and serogroup Y (adjusted odds ratio 4.78; 95% confidence interval 2.61–8.75). Age showed a steep gradient: compared with infants younger than 1 year, odds of pneumonia increased progressively and were highest in adults 65 years or older (adjusted odds ratio 24.1; 95% confidence interval 8.9–65.5; *p* < 10^−9^). No clonal complex remained significantly associated after adjustment.

Sepsis or shock remained associated with serogroup W (adjusted odds ratio 1.89; 95% confidence interval 1.39–2.58) and Y (adjusted odds ratio 2.53; 95% confidence interval 1.78–3.60) and with older age (65 years or older: adjusted odds ratio 4.78; 95% confidence interval 3.68–6.21). cc11 was also independently associated with sepsis or shock (adjusted odds ratio 1.98; 95% confidence interval 1.45–2.69).

Meningeal presentation was associated with serogroup B, but all other serogroups were less likely to present with meningitis. For example, serogroup W (adjusted odds ratio 0.30; 95% confidence interval 0.22–0.40) and Y (adjusted odds ratio 0.28; 95% confidence interval 0.20–0.39) had substantially reduced odds of meningeal involvement compared with B. Older adults had much lower odds (65 years or older: adjusted odds ratio 0.19; 95% confidence interval 0.15–0.24), whereas differences across paediatric and adolescent age groups were modest.

Finally, arthritis was independently associated with serogroup C (adjusted odds ratio 4.19; 95% confidence interval 2.43–7.24), W (adjusted odds ratio 3.55; 95% confidence interval 2.05–6.13) and X (adjusted odds ratio 6.17; 95% confidence interval 1.94–19.58). Odds also increased with age, particularly from 25 years onwards, although confidence intervals were wide.

### 3.9. Antimicrobial Susceptibility

Minimum inhibitory concentration data were available for 3437 isolates for penicillin G, 3409 for amoxicillin, 3436 for cefotaxime, 3437 for rifampicin, 3433 for ciprofloxacin and 773 for azithromycin. Overall proportions of non-susceptible isolates among those tested were as follows.

- Penicillin G: 37.7%.
- Amoxicillin: 37.7%.
- Cefotaxime: 0.6%.
- Rifampicin: 1.5%.
- Ciprofloxacin: 0.5%.
- Azithromycin: 0.9%.

Consistent with the rarity of resistance to these agents in invasive meningococci, non-susceptibility remained uncommon for cefotaxime, rifampicin, ciprofloxacin and azithromycin, and no consistent clinical pattern emerged for these molecules. In contrast, penicillin G showed substantial variability across clinical and microbiological categories and was therefore analysed in more detail.

### 3.10. Determinants of Penicillin G Non-Susceptibility (Multivariable Analysis)

A first multivariable logistic regression model including age group, sex, serogroup and clonal complex, based on 3437 isolates with MIC data, identified strong independent predictors of penicillin G non-susceptibility. Determinants of penicillin G non-susceptibility are summarised in Table 3. In the model adjusted for demographics and microbiological characteristics, penicillin G non-susceptibility was strongly associated with serogroup and clonal complex, whereas age 65 years or older was not associated with non-susceptibility. Using serogroup B as a reference, serogroup Y was associated with lower odds of non-susceptibility, approximately 0.5, whereas other serogroups did not differ significantly. With clonal complex Other or non-assigned as a reference, non-susceptibility was positively associated with cc32, cc9316 and cc461 and negatively associated with cc11, cc23 and cc269, in line with known MIC distribution profiles of hyperinvasive meningococcal lineages in Europe.

A second model including clinical presentations, namely sepsis or shock, meningitis, bacteraemic pneumonia and abdominal form, in addition to age group, sex, serogroup and clonal complex, showed that only sepsis or shock remained independently associated with penicillin G non-susceptibility (adjusted odds ratio 1.68; 95% confidence interval 1.41–2.00). The weak associations observed in univariable analyses for meningitis, with slightly more non-susceptible isolates, and pneumonia, with slightly fewer, disappeared after adjustment for serogroup and clonal complex, indicating that microbiological factors rather than clinical phenotype primarily drive penicillin MIC patterns.

## 4. Discussion

This study extended our previous nationwide analysis of IMD cases from 2015 to 2022 that documented an increase in atypical clinical presentations in France, particularly bacteraemic pneumonia and abdominal forms [21].

By extending surveillance to 2014–2025 and integrating MIC data and multivariable modelling, the present study provides a broader and more mechanistic understanding of IMD epidemiology in France. Three successive periods can now be distinguished: a pre-NPI phase (2014–2015 to 2019–2020), a NPI phase (2020–2021 to 2021–2022) and a post-NPI phase (2022–2023 to 2024–2025). Framed within this extended timeline, the changes observed since 2022 represent not only a pandemic rebound but the continuation of a decade-long reconfiguration of clinical and microbiological patterns.

Adjusted analyses clarify the respective contributions of age, serogroup and clonal complex to clinical phenotype.

Serogroups W and Y independently displayed a strong propensity to cause bacteraemic pneumonia and abdominal presentations, supporting the hypothesis of lineage-specific clinical tropism previously suggested in England [18]. The Swedish report also suggests that isolates belonging to MenW/cc11 “2013 strain” were associated with atypical presentations, including gastrointestinal and respiratory symptoms, a low frequency of meningitis and increased disease severity, particularly among older adults [15]. Age emerged as an equally important determinant: odds of meningococcal pneumonia increased dramatically with age, whereas abdominal presentations peaked in adolescents and young adults and meningitis remained concentrated among infants and young children.

Clonal complexes added further resolution. cc11 retained its historical association with abdominal presentations and sepsis/shock. Meanwhile, cc9316, rare before 2020, has become the predominant W lineage in France since 2023–2024 [27], with clear age-related differences relative to cc11. In our data, abdominal forms also increased among W/cc9316 infections, suggesting that emerging lineages may now contribute to phenotypes previously attributed to cc11.

Lineage-specific clinical tropism is biologically plausible but remains incompletely understood. Potential mechanisms include differences in capsule expression, lipooligosaccharide immunotypes, inflammatory potential, complement evasion or tissue tropism mediated by adhesins and invasion factors, which may vary across hyperinvasive lineages such as cc11 or emerging complexes like cc9316 [28,29,30]. Although causal mechanisms cannot be inferred from surveillance data, the consistency of lineage-associated phenotypes supports further genomic and functional investigations.

Several limitations should be considered when interpreting these findings.

Observed increases in atypical presentations may partly reflect changes in clinician awareness, reporting practices or diagnostic vigilance over time, particularly for extra-meningeal forms that are less classically associated with invasive meningococcal disease. However, in France, clinical information is collected using a standardised national notification form and systematically reviewed at microbiological confirmation, which limits, without eliminating, heterogeneity across centres and periods. Importantly, the post-2022 rise in atypical forms coincided with major shifts in circulating serogroups and clonal complexes, supporting a substantive epidemiological contribution beyond surveillance artefacts alone.

Because invasive meningococcal disease frequently involves multiple organ systems, clinical presentations were analysed as non-exclusive entities. Accordingly, adjusted odds ratios should be interpreted as the independent association with a given presentation rather than as mutually exclusive phenotypes. While co-occurrence may attenuate apparent specificity, this approach reflects real-world clinical complexity and facilitates syndromic recognition rather than rigid categorisation.

Early outcome data were not available for all cases, which may introduce selection bias if completeness differed by age, severity or reporting centre. To mitigate this limitation, multivariable analyses of early mortality were restricted to cases with documented outcomes and adjusted for major demographic and clinical determinants. These findings should therefore be interpreted as correlates of early death within the documented-outcome subset rather than as causal effects.

Clinical phenotype likely reflects a combination of host factors, such as age-related immunity, comorbidities and healthcare-seeking pathways, and bacterial determinants including serogroup and lineage. The absence of detailed comorbidity data is an inherent limitation of surveillance systems and may contribute to residual confounding, particularly among older adults with pneumonia. Nevertheless, the persistence of serogroup- and lineage-specific associations after adjustment for age supports a contribution of bacterial population structure beyond host factors alone.

Beyond their epidemiological significance, these findings have important implications for clinical practice.

Abdominal presentations independently doubled the odds of death within 72 h, consistent with their well-documented tendency to mimic acute surgical conditions and delay antimicrobial treatment [9]. From a clinical perspective, this finding supports considering IMD in the differential diagnosis of intense febrile abdominal syndromes, particularly in adolescents and young adults, and prioritising early blood cultures. When empirical antimicrobial therapy is initiated for suspected intra-abdominal sepsis, awareness of IMD may help ensure timely administration of appropriate systemic therapy and prompt escalation in case of clinical severity.

In contrast, bacteraemic pneumonia did not show increased adjusted mortality despite higher crude fatality, a discrepancy that likely reflects both the age distribution of affected individuals and substantial under-recognition [31,32]. Even when recognised early as a form of IMD, the diagnosis of meningococcal pneumonia is challenging [33,34]. This discrepancy, therefore, indicates that the excess crude mortality observed in pneumonic presentations is largely driven by confounding factors, particularly age and lineage distribution, rather than by pneumonia per se. Our study included only cases that were confirmed by culture and/or PCR in the blood. Capturing the whole burden of meningococcal pneumonia may require including pneumonia cases with respiratory samples that are dominated by capsulated meningococci of 10^6^ CFU/mL or greater [35].

The strong co-occurrence between atypical forms and sepsis/shock, including 96% of bacteraemic pneumonias and 77% of abdominal presentations, has major clinical implications, as blood cultures therefore appear to be a highly sensitive tool for diagnosing IMD. Abdominal presentations are often characterised by intense pain and may mimic acute surgical abdomens, leading to diagnostic delay and unnecessary surgical exploration in up to 10–15% of cases [9]. In this context, IMD is rarely considered as a differential diagnosis in adolescents and young adults with febrile abdominal pain, and blood cultures are not systematically prioritised before surgical decision-making. In contrast, although French guidelines recommend obtaining blood cultures in hospitalised or severe community-acquired pneumonia, they are frequently omitted in routine practice when presentations appear typical [36]. Overall, blood cultures represent an already available but underexploited diagnostic tool that may facilitate earlier recognition of IMD in misleading abdominal or respiratory presentations. These findings suggest that updating clinical training and decision pathways may be essential to ensure timely recognition and treatment of these now-common atypical forms.

*N. meningitidis* remains susceptible to the antibiotic of interest, although penicillin G non-susceptibility is increasing worldwide [37,38,39,40]. This choice is further supported by national surveillance data showing high and increasing rates of penicillin G non-susceptibility, particularly among serogroup B and W isolates, whereas resistance to third-generation cephalosporins remains absent [41]. Yet no clinical presentation was independently associated with penicillin non-susceptibility, suggesting that clinical forms may be driven predominantly by host and lineage-related factors rather than by beta-lactam MIC patterns. Corporately, no association was found between reduced penicillin susceptibility and fatal outcome of IMD in England and Wales [42].

This recent evolution of IMD has driven the recent changes in meningococcal vaccination policy in France. In January 2025, mandatory vaccination against serogroup B and quadrivalent ACWY was implemented in infants under 2 years of age. In addition, a booster dose of MenACWY was recommended for adolescents aged 11–14 years, with a catch-up opportunity up to 24 years of age, and MenB vaccination was made available and reimbursed for adolescents and young adults aged 15–25 years [43,44]. They underscore the need for continued, fine-grained surveillance of IMD incidence, serogroup distribution and clinical manifestations to evaluate the population-level impact of these vaccination strategies and to detect potential shifts in disease presentation over time. Because abdominal and pneumonic presentations were strongly associated with serogroups W and Y, changes in the circulation of these serogroups and their lineages following MenACWY implementation may also modify the clinical spectrum of IMD. Conversely, broader MenB vaccination could influence the residual burden of classical meningeal/septic presentations in younger age groups. Our findings, therefore, provide a baseline to monitor whether vaccination-driven shifts in serogroup and lineage distribution translate into changes in clinical presentation patterns over time.

Some elements are context-specific to France, including the organisation of NRC-based surveillance and the timing and scope of recent vaccination policy changes. However, the main age gradients and the serogroup- and lineage-associated patterns of atypical presentations are likely generalisable to settings where similar meningococcal populations circulate, particularly in Europe.

## 5. Conclusions

This nationwide analysis of more than a decade demonstrates that atypical clinical presentations of invasive meningococcal disease are no longer exceptional in France.

Together, our findings highlight the need to broaden clinical awareness beyond classical meningitis and septic shock, reinforce blood testing by culture and PCR and sustain integrated clinical, microbiological and genomic surveillance. Such efforts are essential to inform vaccination strategies, guide antimicrobial stewardship and anticipate further shifts in the clinical spectrum of IMD.

## Figures and Tables

**Table 1:** Baseline characteristics of invasive meningococcal disease cases, France, 2014–2025.

		n	%
Age group (years)	<1	558	12.9
	1–4	452	10.4
	5–9	169	3.9
	10–14	152	3.5
	15–19	465	10.7
	20–24	393	9.1
	25–44	545	12.6
	45–64	660	15.2
	≥65	933	21.6
	Missing age	1	0.0
Serogroup	B	2067	47.8
	C	565	13.1
	W	846	19.5
	Y	780	18.0
	Other	48	1.1
Clonal complex	Other/NA	1241	28.7
	cc11	928	21.4
	cc9316	349	8.1
	cc32	684	15.8
	cc23	566	13.1
	cc269	149	3.4
	cc162	106	2.4
	cc213	209	4.8
	cc461	96	2.2
Clinical presentation	Sepsis/Shock	2645	61.1
	Meningeal	2375	54.9
	Bacteraemic Pneumonia	334	7.7
	Abdominal Form	345	8.0
	Arthritis	258	6.0
	Epiglottitis	20	0.5
	Cardiac form	38	0.9
Early outcome (all cases)	Survived > 72 h	4000	92.4
	Early death ≤ 72 h	328	7.6

**Table 2:** Multivariable logistic regression models for atypical clinical presentations (abdominal form, bacteraemic pneumonia and arthritis).

Outcome	Variable	OR	IC95_Inf	IC95_Sup	*p*
Abdominal form	Age ≥ 65 years	1.04	0.67	1.62	0.855
	Serogroup C	0.57	0.3	1.06	0.0756
	Serogroup W	2.27	1.35	3.83	0.00204
	Serogroup Y	2.92	1.79	4.76	1.73 × 10^−5^
	Serogroup X	0.95	0.12	7.51	0.963
	Serogroup Other	1.4	0.48	4.07	0.534
	cc11	1.77	1.07	2.94	0.0273
	cc9316	1.38	0.82	2.34	0.225
	cc32	1.04	0.67	1.62	0.861
	cc23	0.42	0.25	0.7	0.000855
	cc269	0.85	0.38	1.92	0.696
	cc162	0.54	0.16	1.77	0.308
	cc213	1.7	0.95	3.04	0.0728
	cc461	1.04	0.4	2.68	0.942
Bacteraemic pneumonia	Age ≥ 65 years	24.38	8.92	66.64	4.76 × 10^−10^
	Serogroup C	1.15	0.52	2.51	0.73
	Serogroup W	5.1	2.66	9.75	8.71 × 10^−7^
	Serogroup Y	4.73	2.59	8.65	4.56 × 10^−7^
	Serogroup X	1.74	0.2	14.95	0.616
	Serogroup Other	6.36	2.16	18.7	0.000779
	cc11	1.07	0.64	1.8	0.794
	cc9316	0.69	0.39	1.21	0.193
	cc32	0.75	0.34	1.64	0.469
	cc23	1.14	0.75	1.74	0.535
	cc269	0.3	0.04	2.32	0.249
	cc162	0.85	0.19	3.91	0.838
	cc213	0.5	0.11	2.23	0.36
	cc461	1.34	0.37	4.82	0.657
Arthritis	Age ≥ 65 years	2.55	1.4	4.67	0.00233
	Serogroup C	4.2	2.44	7.25	2.41 × 10^−7^
	Serogroup W	3.56	2.06	6.15	5.6 × 10^−6^
	Serogroup Y	1.66	0.86	3.18	0.13
	Serogroup X	6.19	1.95	19.62	0.00197
	Serogroup Other	1.75	0.52	5.92	0.369
	cc11	0.66	0.41	1.04	0.0745
	cc9316	0.63	0.35	1.15	0.134
	cc32	1.03	0.61	1.75	0.901
	cc23	1.28	0.69	2.36	0.434
	cc269	0.87	0.34	2.26	0.778
	cc162	1.41	0.54	3.72	0.484
	cc213	1.01	0.44	2.33	0.981
	cc461	0.25	0.03	1.94	0.187

**Table 3:** Determinants of penicillin G non-susceptibility among invasive meningococcal disease isolates.

Model	Variable	OR	IC95_Inf	IC95_Sup	*p*
Model 1: demographics + microbiology	Age ≥ 65 years	0.95	0.74	1.23	0.723
	Serogroup C	0.7	0.5	0.97	0.0318
	Serogroup W	0.82	0.6	1.11	0.193
	Serogroup Y	0.33	0.24	0.47	5.38 × 10^−10^
	Serogroup X	0.42	0.15	1.17	0.0964
	Serogroup Other	1.05	0.58	1.92	0.863
	cc11	0.36	0.26	0.49	7.72 × 10^−11^
	cc9316	4.99	3.54	7.04	4.52 × 10^−20^
	cc32	2.76	2.24	3.41	2.48 × 10^−21^
	cc23	0.48	0.32	0.71	0.000292
	cc269	0.3	0.19	0.47	1.06 × 10^−7^
	cc162	0.49	0.31	0.77	0.00201
	cc213	1.14	0.84	1.55	0.412
	cc461	4.36	2.65	7.17	6.87 × 10^−9^
Model 2: +clinical forms	Sepsis/septic shock	1.68	1.41	2.0	4.46 × 10^−9^
	Meningeal presentation	0.97	0.81	1.16	0.706
	Bacteraemic pneumonia	1.11	0.82	1.51	0.495
	Abdominal form	1.13	0.87	1.47	0.374
	Age ≥ 65 years	0.8	0.61	1.04	0.0954
	Serogroup C	0.7	0.5	0.98	0.0361
	Serogroup W	0.74	0.54	1.01	0.0541
	Serogroup Y	0.29	0.2	0.41	5.6 × 10^−12^
	Serogroup X	0.43	0.15	1.2	0.106
	Serogroup Other	0.97	0.53	1.78	0.924
	cc11	0.33	0.24	0.45	3.2 × 10^−12^
	cc9316	4.95	3.5	7.0	1.53 × 10^−19^
	cc32	2.83	2.29	3.5	7.07 × 10^−22^
	cc23	0.48	0.32	0.71	0.000284
	cc269	0.29	0.18	0.45	6.76 × 10^−8^
	cc162	0.47	0.3	0.74	0.00124
	cc213	1.16	0.85	1.58	0.359
	cc461	4.47	2.71	7.37	4.47 × 10^−9^

## Data Availability

The data presented in this study originate from the French National Reference Centre for Meningococci and *Haemophilus influenzae* and are part of the national surveillance system for invasive meningococcal disease. Due to legal and ethical restrictions related to data protection regulations and the mandate of the National Reference Centre, the data are not publicly available. Aggregated data supporting the findings of this study may be made available from the corresponding authors upon reasonable request, subject to approval by the National Reference Centre and compliance with applicable regulations.
